# Trends in organic peroxide (ROOR) formation in the reactions of C1–C4 alkyl peroxy radicals (RO_2_) in gas

**DOI:** 10.1039/d5sc03559g

**Published:** 2025-08-13

**Authors:** Barbara Nozière

**Affiliations:** a Royal Institute of Technology (KTH), Department of Chemistry 114 28 Stockholm Sweden noziere@kth.se

## Abstract

Organic peroxy radicals (RO_2_) are important intermediates in aerobic systems such as Earth's atmosphere. The existence of a channel producing dialkyl peroxides (ROOR) in their self- and cross-reactions (*i.e.*, between the same or different radicals) has long been debated and considered a theoretical “key problem in the atmospheric chemistry of peroxy radicals”. Over the past decade, observations have suggested that this channel could be an important source of condensable compounds and, ultimately, new aerosol particles in Earth's atmosphere. However, experimental evidence for specific RO_2_ reactions is scarce. In this work, the formation of ROOR in the self- and cross-reactions of eight RO_2_ (CH_3_O_2_, ^13^CH_3_O_2_, CD_3_O_2_, C_2_H_5_O_2_, 1- and iso-C_3_H_7_O_2_, 1- and *tert*-C_4_H_9_O_2_) could be observed by modifying the ionisation conditions on a proton transfer mass spectrometer. The ROOR formation channel was confirmed to be in competition with the other product channels rather than precede them. For six of the RO_2_ studied, the branching ratio, *γ*, for the ROOR channel of the self-reaction was quantified relative to these other channels. The results allowed for the first time to identify some trends in *γ* with respect to the RO_2_ structure: *γ* decreases with increasing RO_2_ chain length for the linear/primary radicals, ranging from (14.1 ± 7)% for CH_3_O_2_ to (1.1 ± 0.5)% for 1-C_4_H_9_O_2_, while branched radicals exhibit much higher *γ* than their linear counterparts, with *γ* = (17.2 ± 8.6)% for iso-C_3_H_7_O_2_ and (46.6 ± 23.2)% for *tert*-C_4_H_9_O_2_. The formation of ROOR products from RO_2_ reactions in the atmosphere should thus be strongly dependent on the RO_2_ structure.

## Abstract 2

The formation of organic peroxides, ROOR, was studied from eight organic peroxy radicals (RO_2_). The formation branching ratio obtained for six of these compounds varied widely with the structure of the RO_2_.

## Graphical abstract

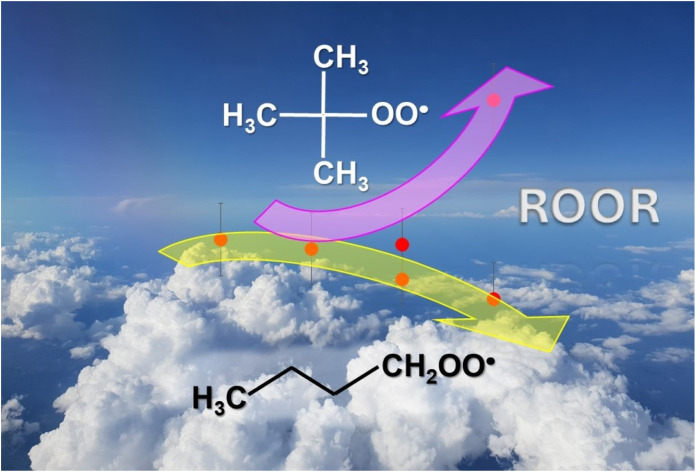

## Introduction

Organic peroxy radicals (RO_2_) are important intermediates in the oxidation of organic compounds in most aerobic chemical systems, such as biochemistry,^1,2^ chemical and food industry,^3,4^ low-temperature combustion,^5^ and Earth's atmosphere.^6,7^ Their main fates in most systems are reactions with other compounds or radicals, but their self-reactions and cross-reactions (*i.e.*, reactions between the same or different RO_2_) can have non-negligible impacts in laboratory investigations. For most RO_2_ radicals, the self-reaction is thought to involve at least two competing product channels:^8^

1a RO_2_ + RO_2_ → ROH + R–H=O + O_2_*α*

1b → 2 RO + O_2_*β*,

where ROH, R–H=O represent the alcohol and carbonyl products of RO_2_, RO the corresponding alkoxy radical, and *α* and *β* the branching ratios of channels (1a) and (1b), respectively. Some early works also reported the existence of a minor channel producing a peroxide: ROOR:^9^

1c RO_2_ + RO_2_ → ROOR + O_2_*γ*.

However, the branching ratio for this channel, *γ*, was estimated to be small: *γ*(CH_3_O_2_) ≤6%, *γ*(C_2_H_5_O_2_) ≤6%, *γ*(HOCH_2_CH_2_O_2_) ≤2%, and *γ*(*tert*-C_4_H_9_O_2_) ≤12%.^9^ For decades, no other study reported the observation of this channel and the latter was ruled out as negligible.^6,9^ Its existence was also difficult to explain theoretically and this channel was referred to as one of the “two key problems in the atmospheric chemistry of peroxy radicals”.^10^ Over the past decade, laboratory investigations^11,12^ and atmospheric observations^13^ have reported the presence of “highly oxygenated molecules” (HOMs) in the gas phase, which systematically include “dimers” (*i.e.*, compounds having twice the number of C-atoms than their precursors). These compounds are expected to play important roles in the formation of new aerosol particles in the atmosphere. The dimers have been attributed to the self- and cross-reactions of RO_2_,^13,14^ and these observations reignited the interest for this potential third channel of RO_2_ + RO_2_. Recent theoretical studies revealed new information on these reactions^15–20^ such as explaining the occurrence of the third channel by intersystem crossing^20^ and evidencing a fourth channel producing esters or ethers with complex RO_2_.^21,22^ However, experimental data remain scarce. In recent years, the branching ratio *γ* has been quantified for the self-reactions of only four radicals (C_2_H_5_O_2_,^23^ CH_2_(OH)CH_2_O_2_,^24^ CH_3_C(O)CH_2_O_2_,^11,25^ and C_3_H_7_O_2_),^26^ which does evidence any trend on how *γ* might vary with the RO_2_ structure.

The present work investigates the formation of ROOR products in the self-reaction of eight RO_2_ (CH_3_O_2_, ^13^CH_3_O_2_, CD_3_O_2_, C_2_H_5_O_2_, 1- and iso-C_3_H_7_O_2_, 1-C_4_H_9_O_2_, *tert*-C_4_H_9_O_2_) and in the cross-reactions of CH_3_O_2_ with C_2_H_5_O_2_ and iso-C_3_H_7_O_2_. In all experiments, radicals and products were monitored with a proton transfer chemical ionization mass spectrometer (CIMS), in which the ionization conditions were modified to enable ROOR detection. After checking the occurrence of the third channel in all of these reactions, the formation kinetics of ROOR were investigated, and the branching ratio *γ* was quantified.

## Experimental section

The experimental setup has been described in previous works.^27,28^ It consists of a vertical quartz reactor of total length *L* = 120 cm, in which organic peroxy radicals, RO_2_, are produced photolytically by flowing a gas mixture through an irradiation window, corresponding to 2–8 s of residence time (see the “Radical production” section). After passing the irradiation window, the gas mixture flows in the dark, allowing further reactions to occur. In the present study, this part corresponded to reaction times between 0.5 and 10 s, which was achieved by moving the position of the irradiation window in the reactor. At the exit of the reactor, a small fraction (<10%) of the reaction mixture was sampled into a CIMS for analyses (see the “Detection” section). In this work, two nearly-identical reactors were used, with different internal diameters: *d* = 3 and 5 cm. The bath gas was synthetic air with a mass flow of 3.0 sLm, and the experiments were performed slightly below atmospheric pressure (*P* = 0.85–0.95 atm) and at room temperature (*T* = 300 ± 4 K). The radical precursors were introduced into the main gas flow by bubbling a small flow of N_2_ through the pure liquids and adding them to the reactor after a dilution loop. A list of the experiments performed in this work is given in Table S1 of the SI.

### Radical production

The radicals were generated by the photolysis of iodinated precursors in a gas mixture flowing through an irradiation window. This irradiation window was surrounded by four narrow-band UV-C lamps (TUV 36W SLV/6; Phillips) emitting at *λ* = 254 nm. For instance, the RO_2_, CH_3_O_2_, was produced by:

2 CH_3_I + *hν* → CH_3_ + I

3 CH_3_ + O_2_ + M → CH_3_O_2_ + M.

Note that the generation of I-atom led to side-reactions:^29^

4 CH_3_O_2_ + I → CH_3_O_2_ I,

5 CH_3_O_2_I + I → CH_3_O_2_ + I_2_.

However, these fast reactions had negligible effects on most of the RO_2_ studied in this work, except *tert*-C_4_H_9_O_2_. The initial concentration of RO_2_ in the different reactions was estimated to be between 1.5 × 10^10^ cm^−3^ (for 1-C_4_H_9_O_2_) and 8 × 10^13^ cm^−3^ (for *tert*-C_4_H_9_O_2_). Note that precise knowledge of the radical concentration was not necessary in this work.

### Detection and quantification of radicals and products with the CIMS

This section only describes the general features of the detection of gas-phase compounds with the CIMS. The specific question of the ionization of ROOR is discussed in the “Results” section. Gas-phase compounds (“A” in reaction (6) below), including radicals and stable products, were ionized by proton transfer with the water clusters, (H_2_O)_*n*_H^+^, and detected with the CIMS:^27,30–32^

6 A + (H_2_O)_*n*_H^+^ → A(H_2_O)_*n*−*m*_H^+^ + *m*H_2_O.

Under the ionization conditions used in this study, the most abundant water/proton clusters were (H_2_O)_3_H^+^ (*m*/*z* 55) and (H_2_O)_4_H^+^ (*m*/*z* 73) rather than H_3_O^+^. Thus, a compound of mass M was detected by its ion products at *m*/*z* M + 19, M + 37, and M + 55. The ion masses at which the radicals and products were monitored in this study are listed in Table S2.

In this work, the branching ratio *γ* for peroxide formation was quantified relative to the branching ratio *α* of channel (1a) or, in the case of *t*-C_4_H_9_O_2_, to channel (1b) producing acetone. This quantification required the determination of the absolute concentration of all the compounds involved (*i.e.*, the alcohols, acetone, and ROOR), thus that of their detection sensitivity, *S*^o^ (Hz ppb^−1^). The detection sensitivities for methanol, ethanol, 1- and 2-propanol, 1-butanol, acetone, and di-*tert*-butyl peroxide were calibrated within ±30% using reference standards. The results are presented in Fig. S1 and showed that, within a class of compounds, *S*^o^ decreases exponentially with the number of C-atoms and is smaller for the substituted compounds than for their linear counterparts. These trends are identical to those reported previously for a range of RO_2_.^33^ Therefore, for the ROOR, for which a standard was not available *S*^o^ was estimated assuming the same trends than for the alcohols and RO_2_. In practice, this meant that *S*^o^(ROOR) was estimated by dividing *S*^o^ for the corresponding alcohol by a factor of ∼3. This led to ±50% of uncertainties on the estimated *S*^o^ values because of the wide range of values included in the extrapolation. These uncertainties propagated to the determination of the absolute concentrations for the ROOR in the experiments (except di-*tert*-butyl peroxide) and to the product ratios used to determine the branching ratio *γ*.

### Chemicals

HiQ Synthetic air 5.0 was obtained from Linde Gas. NO (special mixture: 200 ppm in N_2_), was purchased from Air Liquide. Iodomethane (CAS 74-88-4), 99.5%, was procured from Chemtronica. Iodoethane (CAS 75-03-6), 99%, and 1-iodobutane (CAS 542-69-8), 98%, were obtained from ACROS/Fisher. The following chemicals were purchased from MilliporeSigma (formerly Sigma-Aldrich): iodomethane-*D*_3_ (CAS 865-50-9), ≥99.5%; ^13^C-iodomethane (CAS 4227-95-6), 99%; 1-iodopropane (CAS 107-08-4), ≥98.5%; 2-iodopropane (CAS 75-30-9), 99%; 2-iodo 2-methylpropane (CAS 558-17-8), 95%; di-*tert*-butyl peroxide (CAS 110-05-4), 98%; methanol (CAS 67-56-1), 99.8+%; ethanol (CAS 64-17-5), 99+%; 1-propanol (CAS 71-23-8), ≥99.9%; iso-propanol (CAS 67-63-0), 99.9%; 1-butanol (CAS 71-36-3), ≥99.%; acetone (CAS 67-64-1), 99.0%.

## Results and discussion

### Favoring proton transfer over fragmentation in the detection of organic peroxides

Studies have reported that the ionisation of organic peroxides (ROOR) and hydroperoxides (ROOH) by proton transfer mass spectrometry proceeds exclusively by fragmentation (channel (7a) below) rather than by proton transfer (channel (7b)):^34^

7a ROOR (or ROOH) + H_3_O^+^ → RO^+^ + neutral fragments,

7b ROOR (or ROOH) + H_3_O^+^ → (ROOR)H^+^ (or (ROOH)H^+^) + H_2_O.

The ion fragment RO^+^ is an isomer of the analog carbonyl ion (*e.g.*, CH_3_O^+^ and (HCHO)H^+^ in the CH_3_O_2_ system; C_2_H_5_O^+^ and (CH_3_CHO)H^+^ in the C_2_H_5_O_2_ system). Since carbonyl compounds are usually much more abundant than ROOR, the fragmentation precluded the detection of organic peroxides and hydroperoxides.

**Fig. 1:**
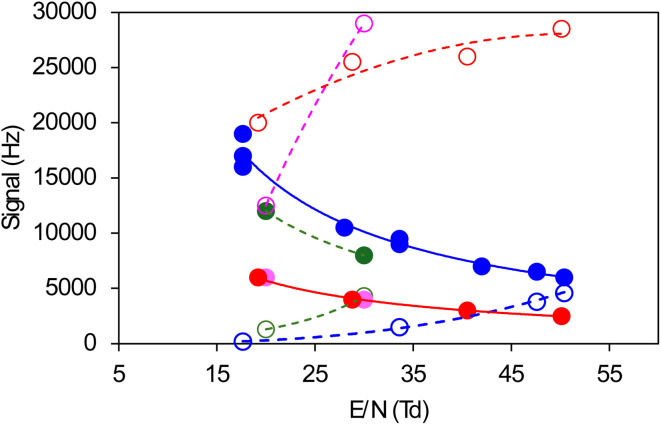
Fragmentation and proton transfer channels for H_3_COOCH_3_ in a CIMS as a function of the ionization conditions (energy, E/N (in Td), and drift tube pressure). Open symbols = fragmentation channel monitored at *m*/*z* 31. Solid symbols = proton transfer channel, sum of *m*/*z* 81 and 99. Colors correspond to different pressures in the drift tube: blue = 20 torr; green = 15 torr; pink = 10 torr; red = 5 torr.

In this work, observing a standard of di-*tert*-butyl peroxide (*t*-C_4_H_9_OOt-C_4_H_9_) with the ionisation conditions used in our previous works to detect RO_2_ (ref. 28 and 31) (*i.e.*, a drift tube pressure of *P*_drift_ = 10 torr and electrical energy of E/N ∼45 Td) led to distinct ion signals at *m*/*z* 165 and 183. These corresponded to the ions expected from proton transfer (Table S2) and indicated that this compound was, in fact, undergoing proton transfer in the CIMS. It was not possible to determine if the main ion for this compound was also undergoing fragmentation because the expected fragment RO^+^, *m*/*z* 73, overlapped with the most intense proton water cluster, (H_2_O)_4_H^+^. Thus, investigation of the proton transfer and fragmentation channels for ROOR was pursued with H_3_COOCH_3_ in the self-reaction of CH_3_O_2_. The masses for the proton transfer and fragmentation ions for H_3_COOCH_3_ were *m*/*z* 81 and 99 (Table S2), and *m*/*z* 31, respectively, which allowed us to monitor both channels separately. Note that, under identical ionization conditions, HCHO was exclusively detected at *m*/*z* 67 and 85 (Table S2) and had a negligible signal at *m*/*z* 31, and therefore did not interfere with the monitoring of the fragment CH_3_O^+^. The proton transfer and fragmentation channels of H_3_COOCH_3_ were then studied by maintaining the experimental conditions unchanged while varying the ionization conditions, mostly the electrical energy E/N (in Td) and drift tube pressure (Fig. 1). The fragmentation channel increased with the energy E/N and decreased with the drift tube pressure. Fragmentation dominated over proton transfer at and below 10 torr. This corresponds to the conditions in most commercial proton transfer mass spectrometers and to the fragmentation of organic peroxides reported in previous studies.^34^ By contrast, proton transfer dominated over fragmentation at and above 15 torr. A drift tube pressure between 15 and 20 torr and an energy E/N between 15 and 35 Td were thus systematically used in this study to ensure that all the peroxides were detected by their proton transfer ions (Table S2).

### Peroxide formation in the self- and cross-reactions of C1–C4 RO_2_

**Fig. 2:**
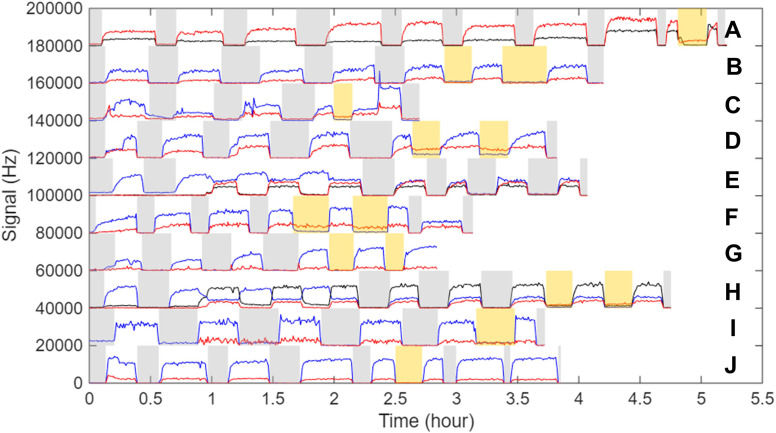
Experimental signals for RO_2_ and ROOR products. Grey areas: lights OFF. Orange areas: NO on. Black lines = CH_3_O_2_. Blue lines = other RO_2_. Red lines = ROOR (and ROOR′). Signals have been scaled for clarity. (A) CH_3_O_2_ and H_3_COOCH_3_ (both x½). (B) CD_3_O_2_ and D_3_COOCD_3_ (both x2). (C) ^13^CH_3_O_2_ and ^13^CH_3_OO^13^CH_3_ (x3). (D) C_2_H_5_O_2_ and C_2_H_5_OOC_2_H_5_ (both x2). (E) C_2_H_5_O_2_, CH_3_O_2_ and C_2_H_5_OOCH_3_. (F) 1-C_3_H_7_O_2_ and 1-C_3_H_7_OOC_3_H_7_ (x3). (G) i-C_3_H_7_O_2_ and i-C_3_H_7_OOC_3_H_7_ (x20). (H) i-C_3_H_7_O_2_, CH_3_O_2_, and i-C_3_H_7_OOCH_3_. (I) 1-C_4_H_9_O_2_ (x2) and 1-C_4_H_9_OOC_4_H_9_ (x19). (J) *t*-C_4_H_9_O_2_ (x1/2) and *t*-C_4_H_9_OOC_4_H_9_.

In this work the self-reaction of eight RO_2_ (CH_3_O_2_, ^13^CH_3_O_2_, CD_3_O_2_, C_2_H_5_O_2_, 1- and iso-C_3_H_7_O_2_, 1-C_4_H_9_O_2_, *tert*-C_4_H_9_O_2_) was studied as well as the cross-reactions of CH_3_O_2_ with C_2_H_5_O_2_ and iso-C_3_H_7_O_2_, producing ten different ROOR and ROOR′. The time profiles of these peroxides, along with those of the RO_2_, are presented in Fig. 2. To confirm the identity of the ROOR and exclude the contribution of pollution or other artefacts on the signals, each experiment included several cycles in which the lights were turned OFF (grey areas in Fig. 2) and at least one cycle with NO being added into the reactor (orange areas in Fig. 2). Studies of the cross-reactions with CH_3_O_2_ also involved several cycles in which the precursor (CH_3_I) was turned ON/OFF. As shown in Fig. 2, the ROOR and RO_2_ signals disappeared when the lights were off or when NO was added, confirming that the ROOR were products of RO_2_ reactions. These cycles also indicated the background signal level to be subtracted when quantifying these compounds.

Observation of these ten different ROOR and ROOR′ confirmed the universality of the peroxide-producing channel in these reactions.

### Testing different formation mechanisms for ROOR

Next, the formation kinetics for ROOR were investigated to determine if this product resulted from the generally assumed mechanism of reaction (1) (three parallel product channels, “Mechanism I”), or potentially from an alternative mechanism.

An alternative mechanism (“Mechanism II”) could be, for instance, the production of ROOR as the sole product in a first step (reaction (8) below), followed by its decomposition into the two other product channels (reaction (9)):

8 RO_2_ + RO_2_ → ROOR + O_2_

9a ROOR → 2 RO

9b ROOR → ROH + R–H=O

To determine which mechanism was taking place, kinetic simulations were performed using CH_3_O_2_ and *t*-C_4_H_9_O_2_ as model RO_2_, and compared with the experimental data (see details in Section S1). These simulations showed that, in all cases, Mechanism I resulted in a product ratio R = [ROH]/[ROOR] and R = [acetone]/[ROOR] not varying significantly over 0–10 s of reaction time (Fig. S2B and S3B–C). By contrast, Mechanism II led to the ratios R increasing by orders of magnitude over the same timescale (Fig. S2 and S3).

Experimental values for R = [ROH]/[ROOR] and R = [acetone]/[ROOR] in each experiment were obtained from the absolute concentrations of the alcohols, acetone, and peroxides. The latter were determined from the respective experimental signals, after subtraction of the background signal obtained in the absence of light. These net signals were then divided by the detection sensitivity, *S*^o^ (Hz ppb^−1^), determined as described in the Experimental section (*i.e.*, by direct calibration for the alcohols, acetone and di-*tert*-butyl peroxide, and by extrapolation from the known *S*^o^ for the other ROOR). These product ratios, R, were then determined at different reaction times by moving the position of the irradiation window in the reactor. The results are presented in Fig. S5 and compared with the kinetic simulations in Fig. S2B, C, S3C and D. Within the uncertainties (estimated to ±50%), these experimental ratios did not vary significantly over 0–10 s of reaction time and, in any case, much less than expected from Mechanism II. These results clearly established that the mechanism governing the self-reaction of RO_2_ was Mechanism I, as generally expected, in which the peroxide ROOR is formed in parallel with the other product channels.

### Quantification of the peroxide yield, *γ*, in the self-reaction of RO_2_

**Fig. 3:**
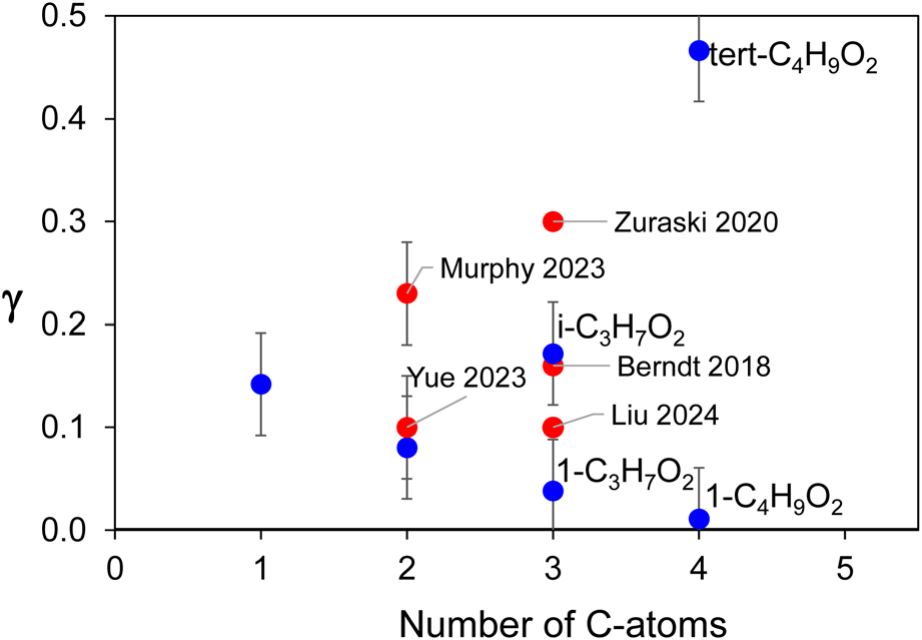
Branching ratio, *γ*, for the formation of ROOR in the self-reactions of RO_2_ studied in this work (blue symbols) and comparison with literature data (red symbols).

Once it was established that ROOR was produced in parallel to channel (1a) and (1b), the branching ratio *γ* was quantified from the product ratios R and the values of *α* or *β* recommended in the literature (see details in Section S2). To validate this approach, it was important to establish first that the product ratios did not vary significantly with the initial concentration of RO_2_ (*i.e.*, between experiments for the same RO_2_) and were mostly controlled by the relative branching ratios. This was verified by performing kinetic simulations with CH_3_O_2_ and *t*-C_4_H_9_O_2_, in which [RO_2_]_o_ was varied by two orders of magnitude (Section S1 and Fig. S2B, S3B and C). In all cases, the product ratio, R, varied only by 20–25%. Thus, this ratio was not expected to vary within a series of experiments, where [RO_2_]_o_ varied by less than one order of magnitude. Relationships determining *γ* from the experimental product ratio, R, and the branching ratios *α* or *β* recommended in the literature were then established (see Section S2 for details). The expressions obtained were, for most RO_2_:10
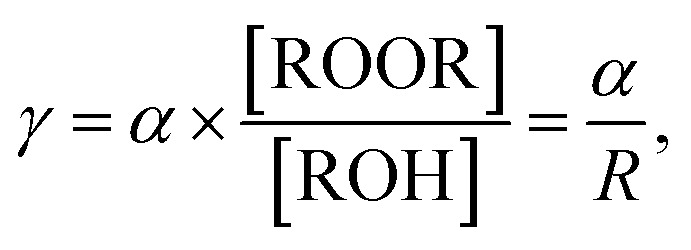
and for *tert*-C_4_H_9_O_2_11
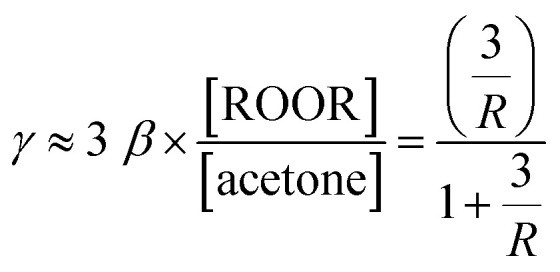
Using the experimental values for the ratio, R, determined above, *γ* was determined for the self-reaction of six of the eight RO_2_ studied (Table 1 and Fig. 3). Note that *γ* was not quantified for CD_3_O_2_ and ^13^CH_3_O_2_ nor for the cross-reactions, mostly because the branching ratios for the other product channels in these reactions are not known, thus precluding the determination of *γ*, even if [ROH] and [ROOR] could be measured. In particular, the cross-reactions had four product channels 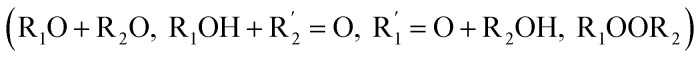, for which none of the branching ratios is known.

**Table 1:** Determination of the branching ratio *γ* from the experimental ratio R and literature values for *α*

Expt. no.	RO_2_	*α*	*R*_obs_	*γ*	Average *γ*
PER1	CH_3_O_2_	0.48^a^	3.3	0.147	
PER2	CH_3_O_2_	0.46^a^	2.8	0.166	
PER3	CH_3_O_2_	0.46^a^	2.7	0.170	
PER4	CH_3_O_2_	0.47^a^	3.0	0.158	
PER5	CH_3_O_2_	0.55^a^	6.7	0.082	
PER6	CH_3_O_2_	0.50^a^	3.9	0.129	
PER7	CH_3_O_2_	0.49^a^	3.5	0.140	**0.141**
PER8	C_2_H_5_O_2_	0.3	3.3	0.091	
PER9	C_2_H_5_O_2_	0.3	3.3	0.091	
PER10	C_2_H_5_O_2_	0.3	4.4	0.071	
PER11	C_2_H_5_O_2_	0.3	4.8	0.068	**0.080**
PER12	iso-C_3_H_7_O_2_	0.44	2.8	0.259	
PER13	iso-C_3_H_7_O_2_	0.44	15.3	0.050	
PER14	iso-C_3_H_7_O_2_	0.44	3.9	0.207	**0.172**
PER15	1-C_3_H_7_O_2_	0.3^b^	5.1	0.055	
PER16	1-C_3_H_7_O_2_	0.3^b^	11.6	0.031	
PER17	1-C_3_H_7_O_2_	0.3^b^	12.6	0.029	**0.038**
PER18	1-C_4_H_9_O_2_	0.3^b^	27.4	0.011	
PER19	1-C_4_H_9_O_2_	0.3^b^	31.0	0.010	
PER20	1-C_4_H_9_O_2_	0.3^b^	25.2	0.012	**0.011**
PER21	*tert*-C_4_H_9_O_2_	—	3.24	0.481	
PER22	*tert*-C_4_H_9_O_2_	—	3.06	0.495	
PER23	*tert*-C_4_H_9_O_2_	—	3.51	0.461	
PER24	*tert*-C_4_H_9_O_2_	—	4.00	0.429	**0.466**

a Calculated from *β* from ref. 9 and R.

b Assumed identical to *α* for C_2_H_5_O_2_.

The uncertainties in the branching ratios *γ* obtained were mostly those on the product ratios R. The latter were, in turn, a combination of the uncertainties on the alcohol (or acetone) and ROOR concentration.

The uncertainties on the absolute concentrations were mostly those on the detection sensitivity, *S*^o^, because the experimental signals were generally measured to ±15%. However, the uncertainties on the ratio R were not the direct sum of those on the alcohol (or acetone) and ROOR concentrations because these partly compensated each other, especially because the detection sensitivity for most ROOR was estimated from that of the alcohols. Hence, the overall uncertainties on R (and, therefore, on *γ*) were assumed to result mostly from those on the detection sensitivities for the ROOR *i.e.*, ±50%. The lack of variation of the ratio, R, with reaction time and the initial radical concentration makes this methodology for quantifying *γ* robust. Relying on concentration ratios also compensated, to a certain extent, for the variability in the ionisation conditions between experiments.

All the branching ratios measured in this work are larger than those recommended in the IUPAC database:^9^ 14.1% (instead of ≤6%) for CH_3_O_2_, 8.0% (instead of ≤6%) for C_2_H_5_O_2_, and 46.6% (instead of ≤2%) for *tert*-C_4_H_9_O_2_. These differences could be attributed to the difficulty in observing and quantifying ROOR compounds with multiple analytical methods.

The branching ratios reported here for six RO_2_ allow, for the first time, to distinguish some trends in the variation of *γ* with the RO_2_ structure. Two trends are visible in Fig. 3: (i) a decrease in *γ* with increase in the number of C-atoms for the linear/primary alkyl RO_2_; (ii) larger *γ* for the branched RO_2_ than for the linear counterparts. Thus, peroxide formation seems to be a minor channel for linear/primary RO_2_ (except perhaps CH_3_O_2_) and mostly significant for substituted ones.

The value of *γ* = 8 ± 4% obtained in this work for the peroxide of C_2_H_5_O_2_ agrees well with the previous determination of *γ* = 10 ± 5%^23^ for this compound (Fig. 3). The values of *γ* = 3.8 ± 1.9% obtained for the peroxide of 1-C_3_H_7_O_2_ and *γ* = 17.2 ± 8.6% for i-C_3_H_7_O_2_ are consistent with *γ* = 10 ± 5% reported previously for a mixture of both compounds.^26^ Larger branching ratios have also been reported for functionalized RO_2_ compared with the corresponding alkyl RO_2_: *γ* = 23 ± 5% for the peroxide of HOCH_2_CH_2_O_2_ (ref. 24), thus larger than for C_2_H_5_O_2_, and *γ* = 16 x2/2% (ref. 11) and 30 × 2/2% (ref. 25) for that of CH_3_C(O)CH_2_O_2_, both larger than for 1-C_3_H_7_O_2_. This comparison shows that, beside substitution, some functionalization also enhances the formation yield of ROOR.

## Conclusions and atmospheric implications

By exploring the self- and cross-reactions of eight RO_2_, we confirmed the general existence of a channel producing ROOR in these reactions. Our experimental data indicate that peroxides are formed in parallel to the other product channels, and do not precede them. The branching ratios, *γ*, measured for six of the RO_2_ studied were all larger than those recommended in databases.^9^ These results reveal some distinct trends in *γ* with radical structure: a decrease with increasing radical size for linear and primary RO_2_, and larger *γ* for branched radicals relative to their linear counterparts. Thus, the formation of ROOR is expected to be small for most linear/primary RO_2_, and mostly significant for branched ones. A comparison of these results with literature data for peroxide formation from functionalized RO_2_ also show that functionalization enhances this channel compared with the linear alkyl analogs.

Because of the relative abundance of CH_3_O_2_ in the atmosphere, the branching ratio *γ* = 14.1 ± 7% reported in this work might result in non-negligible concentration of its peroxide in low-NO_*x*_ regions. Cross-peroxides between CH_3_O_2_ and other RO_2_ would also be favored in such environments. Otherwise, the formation of organic peroxides from RO_2_ self- and cross-reactions in the atmosphere is expected to be mostly important for large (C ≥ 5) and substituted RO_2_ resulting from organic precursors of global importance, such as isoprene and terpenes.^24^ The competition between RO_2_ self- and cross-reactions and their other reactions (with NO, HO_2_…) in the atmosphere is likely to be the main limit for the formation of organic peroxides by these pathways. Other potential formation mechanisms could be considered for these compounds, such as condensed-phase or surface reactions.^35^

## Author contributions

Funding acquisition, methodology, experimental, writing: BN.

## Conflicts of interest

There are no conflicts of interest to declare.

## Supplementary Material

SC-016-D5SC03559G-s001

## Data Availability

The raw data of our experiments is posted on Zenodo at https://doi.org/10.5281/zenodo.16672804 and is freely available. Some of the data supporting the work presented are provided in the SI. See DOI: https://doi.org/10.1039/d5sc03559g.
