# Supplementary material for: Trends in organic peroxide (ROOR) formation in the reactions of C1–C4 alkyl peroxy radicals (RO2) in gas
Source: Chem Sci. 2025 Aug 13;16(36):16590–6. doi: 10.1039/d5sc03559g (PMC12379717; doi:10.1039/d5sc03559g)
Supplement: SC-016-D5SC03559G-s001 [file SC-016-D5SC03559G-s001.pdf]

Supplementary Information (SI) for Chemical Science.
This journal is © The Royal Society of Chemistry 2025

Supplementary Information on

Trends in Organic Peroxide (ROOR) Formation in the Reactions of C1-C4 Alkyl Peroxy Radicals (RO_2) in the gas

By B. Noziere

Contents

Table S1. List of the experiments 2

Table S2: Ion masses (*m/z*) at which compounds were monitored. 3

Figure S1: Detection sensitivities calibrated and estimated in this work. 4

Section S1: Comparison of Mechanism I and II for the formation kinetics for ROOR .5

Table S3: Reactions and rate coefficients in Mechanisms I and II for CH_3O_2. 6

Figure S2: Comparison of mechanisms I and II for CH_3O_2. 7

Table S4: Reactions and rate coefficients in Mechanisms I and II for t-$C_4H_9O_2$. 8

Figure S3: Comparison of Mechanisms I and II for tert-$C_4H_9O_2$..............................9

Figure S4: Effects of side-reactions on the time profiles and product ratios. 10

Figure S5: Evolution of the product ratios for different RO_2 in the experiments.........11

Section S2: Determination of γ from the observed product ratios and literature data for the other channels 12

Figure S6: Results of the kinetic simulations. 14

References...15

Table S1. List of the experiments

Expt. N.	RO_2 -I	Precursor-I, CAS number	Precursor-I concentration (ppm)	RO_2 -II	Precursor-II concentration (ppm)	Pdrift (Torr)	E/N (Td)
PER1	CH_3O_2	CH_3I, 74-88-4	13.7	/	/	20.4	17.5
PER2	CH_3O_2	CH_3I, 74-88-4	13.7	/	/	20.4	17.5
PER3	CH_3O_2	CH_3I, 74-88-4	13.7	/	/	20.5	17.4
PER4	CH_3O_2	CH_3I, 74-88-4	11.4	/	/	20.0	17.8
PER5	CH_3O_2	CH_3I, 74-88-4	12.9	/	/	22.0	21.9
PER6	CH_3O_2	CH_3I, 74-88-4	12.9	/	/	20.0	24.3
PER7	CH_3O_2	CH_3I, 74-88-4	12.9	/	/	14.2	33.8
PER8	$C_2H_5O_2$	C_2H_5I, 75-03-6	5.8	/	/	20.4	17.6
PER9	$C_2H_5O_2$	C_2H_5I, 75-03-6	5.8	/	/	20.1	17.8
PER10	$C_2H_5O_2$	C_2H_5I, 75-03-6	83.4	/	/	19.95	26.8
PER11	$C_2H_5O_2$	C_2H_5I, 75-03-6	7.2	/	/	15.3	36.1
PER12	iso-$C_3H_7O_2$	2-C_3H_7I, 75-30-9	10.5	/	/	19.7	18.07
PER13	iso-$C_3H_7O_2$	2-C_3H_7I, 75-30-9	10.7	/	/	20.3	17.6
PER14	iso-$C_3H_7O_2$	2-C_3H_7I, 75-30-9	7.0	/	/	25.3	14.7
PER15	1-$C_3H_7O_2$	1-C_3H_7I, 107-08-4	14.4	/	/	20.6	17.3
PER16	1-$C_3H_7O_2$	1-C_3H_7I, 107-08-4	2.8	/	/	20.9	24.2
PER17	1-$C_3H_7O_2$	1-C_3H_7I, 107-08-4	2.8	/	/	16.2	30.0
PER18	1-$C_4H_9O_2$	1-C_4H_9I, 542-69-8	8.4	/	/	20.0	17.8
PER19	1-$C_4H_9O_2$	1-C_4H_9I, 542-69-8	7.1	/	/	18.6	26.0
PER20	1-$C_4H_9O_2$	1-C_4H_9I, 542-69-8	7.1	/	/	16.5	29.1
PER21	tert-$C_4H_9O_2$	tert-C_4H_9I, 558-17-8	7.7	/	/	20.3	17.5
PER22	tert-$C_4H_9O_2$	tert-C_4H_9I, 558-17-8	7.7	/	/	20.4	17.5
PER23	tert-$C_4H_9O_2$	tert-C_4H_9I, 558-17-8	3.9	/	/	15.5	31.0
PER24	tert-$C_4H_9O_2$	tert-C_4H_9I, 558-17-8	5.2	/	/	15.5	31.4
PER25	$C_2H_5O_2$	C_2H_5I, 75-03-6	5.8	CH_3O_2	17.1	20.4	17.6
PER26	iso-$C_3H_7O_2$	2-C_3H_7I, 75-30-9	10.5	CH_3O_2	8.6	19.7	18.07
PER27	iso-$C_3H_7O_2$	2-C_3H_7I, 75-30-9	10.7	CH_3O_2	8.6	20.3	17.6
PER28	iso-$C_3H_7O_2$	2-C_3H_7I, 75-30-9	7.0	CH_3O_2	22.8	25.3	14.7
PER29	CD_3O_2	CD_3I, 865-50-9	100.3	/	/	20.2	17.5
PER30	CD_3O_2	CD_3I, 865-50-9	111.1	/	/	20.3	17.5
PER31	CD_3O_2	CD_3I, 865-50-9	101.3	/	/	19.9	17.8
PER32	$^{13}CH_3O_2$	$^{13}CH_3I$, 4227-95-6	17.3	/	/	20.3	17.5
PER33	$^{13}CH_3O_2$	$^{13}CH_3I$, 4227-95-6	67.5	/	/	20.3	17.5

Table S2: Ion masses (*m/z*) at which compounds were monitored.

	RO_2	ROOR	ROOR' with CH_3O_2	ROH	R_{-H}=O
**CH_3O_2** MW = 47.013	84/102	81/99	/	69/87	67/85
**CD_3O_2** MW = 50.032	87/105	105/123*	/	73/91 **CD_3OH 72/90	69/87/105
**$^{13}CH_3O_2$** MW = 48.017	85/103	101/119	/	70/88	68/86/104
**H_3C-CH_2O_2** MW = 61.029	80/98	109/127	95/113	65/83	63/81
**H_3C-CH_2-CH_2O_2** MW = 75.045	94/112	137/155	/	79/97	77/95
**H_3C-CHO_2-CH_3** MW = 75.045	94/112	137/155	109/127	79/97	77/95
**1-$C_4H_9O_2$** MW = 89.060	108/126	165/183	/	93/111	73/91/109
**$(H_3C)_3$-CO_2** MW = 89.060	108/126	165/183	/	/	77/95

*overlapping of ROOR with R=O and RO_2;**the main product observed was CD_3OH not CD_3OD.

Figure S1: Detection sensitivities calibrated and estimated in this work.

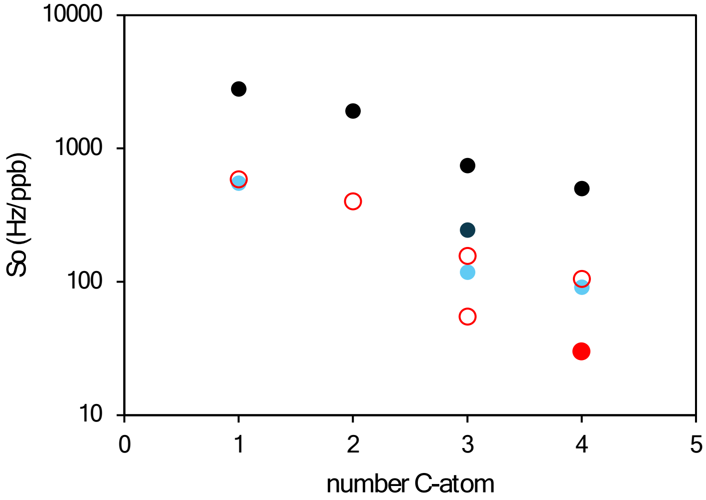

Figure S1: Detection sensitivities, S° (Hz/ppb), calibrated and estimated in this work. Full symbols = calibrations made in this work; black: alcohols; clear blue: RO_2; red: di-tert-butyl peroxide; open symbols = sensitivities estimated for the other ROOR assuming the same variation as for the calibrated compounds, i.e. in practice S°(ROOR) ~ S°(ROH)/ 3. All the detection sensitivities are normalized for P_{drift}= 20 Torr and E/N = 17.5 Td.

Section S1: Comparison of Mechanism I and II for the formation kinetics for ROOR

To determine if mechanism I (three parallel product channels) or mechanisms II (ROOR produced first) is occurring in the self-reaction of RO_2 kinetic simulations were performed with Chemsimul 3.90 and the results were compared with the experimental data. This was performed for CH_3O_2 (model in Table S3 and results in Figure S2) and for t-$C_4H_9O_2$ (model in Table S4 and results in Figure S3). For all RO_2, the simulations show that Mechanism I results in ratios R = [ROH]/[ROOR] and [acetone] / [ROOR] that are nearly constant over 0 – 10 s (Figures S2B and S3B and C) while Mechanism II results in a fast decrease of [ROOR], thus in ratios R increasing strongly over time (Figures S2C and S3D).
For CH_3O_2, comparing these simulations with the experimental ratios (PER4 in Figure S2 and all data in Figure S5) shows that the latter are constant in the experiments, within the ± 50 % uncertainties, thus indicating that Mechanism I is taking place. For t-$C_4H_9O_2$, both the simulations and experimental profiles show that the kinetics is slow (Figure S3A). The concentrations are not evolving much over 0-10 s and are mostly controlled by the concentrations produced in the irradiation window. Note that the kinetic models in the irradiation region are identical to those in Table S3 and S4, except that it starts with the photolysis of the iodinated precursor and that the concentrations of all other compounds are set to 0 initially. Simulations indicate that the photolysis of RO2 and ROOH in the irradiation region are limited and have little impact on the overall kinetics. A comparison of the simulated product ratio in the irradiated region and in reaction region with the experimental ratios (for PER21 in Figure S3A-D and all data in Figure S5) shows that the latter do not vary significantly, within the uncertainties, thus indicating that mechanism I is taking place. For the data of PER21, a small increase in the ratio can be seen at long reactions time in Figure S3C, but this is due to the uncertainties in the concentrations, and is negligible compared to the increase expected from Mechanism II, as shown in Figure S3D.

In addition, to be able to determine γ from these product ratios, it was important to demonstrate that the latter are independent of the initial concentration of RO_2 (i.e. do not vary between experiments). The results in Figure S2B clearly show that this is the case for CH_3O_2, with R varying by less than 25 % while $[CH_3O_2]_o$ s varied by two orders of magnitude. Similar results are obtained for t-$C_4H_9O_2$ (Figure S3B-C), with the ratio varying by about 20 % (mostly in the irradiation region) when $[RO_2]_o$ is varied by two orders of magnitude. This confirms that the product ratios R measured in different experiments, thus with different initial radical concentration, should not vary and can be used to determine γ.

In addition to the analyses that are directly necessary to determine γ in this work, simulations were performed to determine the potential effects of side-reactions on the time profiles and, most importantly, on the product ratios. The results are presented in Figure S4. In the CH_3O_2 system, the impact of CH_3O_2 + HO_2 was found to be small on the time profiles, but negligible on the product ratio. The impact of the reaction with I-atoms were all found to be negligible, which was expected because most I-atoms are entirely consumed less than 1 s outside of the irradiation region. In the t-$C_4H_9O_2$ system, the reactions with I-atoms were also found to have a negligible effect for the same reason as in the CH_3O_2 system. Surprisingly the cross-reaction t-$C_4H_9O_2$ was also found to have a negligible effect, probably because most of the reaction occurs in the irradiation region.

Table S3: Reactions and rate coefficients in Mechanisms I and II for CH_3O_2.

Mechanism I	Mechanism II
$CH_3O_2 + CH_3O_2 \rightarrow CH_3OH + HCHO + O_2$ (1a)	$CH_3O_2 + CH_3O_2 \rightarrow H_3COOCH_3 + O_2$ (8)
$CH_3O_2 + CH_3O_2 \rightarrow 2\ CH_3O + O_2$ (1b)	$H_3COOCH_3 \rightarrow CH_3OH + HCHO + O_2$ (9a)
$CH_3O_2 + CH_3O_2 \rightarrow H_3COOCH_3 + O_2$ (1c)	$H_3COOCH_3 \rightarrow 2\ CH_3O + O_2$ (9b)
$CH_3O + O_2 \rightarrow HCHO + HO_2$ (10)	$CH_3O + O_2 \rightarrow HCHO + HO_2$ (10)
$CH_3O_2 + HO_2 \rightarrow CH_3OOH + O_2$ (11)	$CH_3O_2 + HO_2 \rightarrow CH_3OOH + O_2$ (11)
$HO_2 + HO_2 \rightarrow H_2O2 + O_2$ (12)	$HO_2 + HO_2 \rightarrow H_2O2 + O_2$ (12)
$CH_3O_2 + I \rightarrow CH_3O_2I$ (13)	$CH_3O_2 + I \rightarrow CH_3O_2I$ (13)
$CH_3O_2I + I \rightarrow CH_3O_2 + I_2$ (14)	$CH_3O_2I + I \rightarrow CH_3O_2 + I_2$ (14)
$HO_2 + I \rightarrow HI + O_2$ (15)	$HO_2 + I \rightarrow HI + O_2$ (15)
$k_{1a} = 3.5 \times 10^{-13} \times 0.49$* $s^{-1}\ cm^{-3}$ [1]	$k_8 = 3.5 \times 10^{-13}\ s^{-1}\ cm^{-3}$ [1]
$k_{1b} = 3.5 \times 10^{-13} \times 0.37\ s^{-1}\ cm^{-3}$ [1]	$k_{9a} = 2\ s^{-1}$ *
$k_{1c} = 3.5 \times 10^{-13} \times 0.14$* $s^{-1}\ cm^{-3}$ *	$k_{9b} = 1\ s^{-1}$ *
$k_{10} = 2 \times 10^{-15}\ s^{-1}\ cm^{-3}$ [1]	
$k_{11} = 5.2 \times 10^{-12}\ s^{-1}\ cm^{-3}$ [1]	
$k_{12} = 1.6 \times 10^{-12}\ s^{-1}\ cm^{-3}$ [1]	
$k_{13} = 2 \times 10^{-11}\ s^{-1}\ cm^{-3}$ [2]	
$k_{14} = 1.5 \times 10^{-10}\ s^{-1}\ cm^{-3}$ [2]	
$k_{15} = 4 \times 10^{-14}\ s^{-1}\ cm^{-3}$ [1]	

*estimated in this work.

Figure S2: Comparison of mechanisms I and II for CH_3O_2.

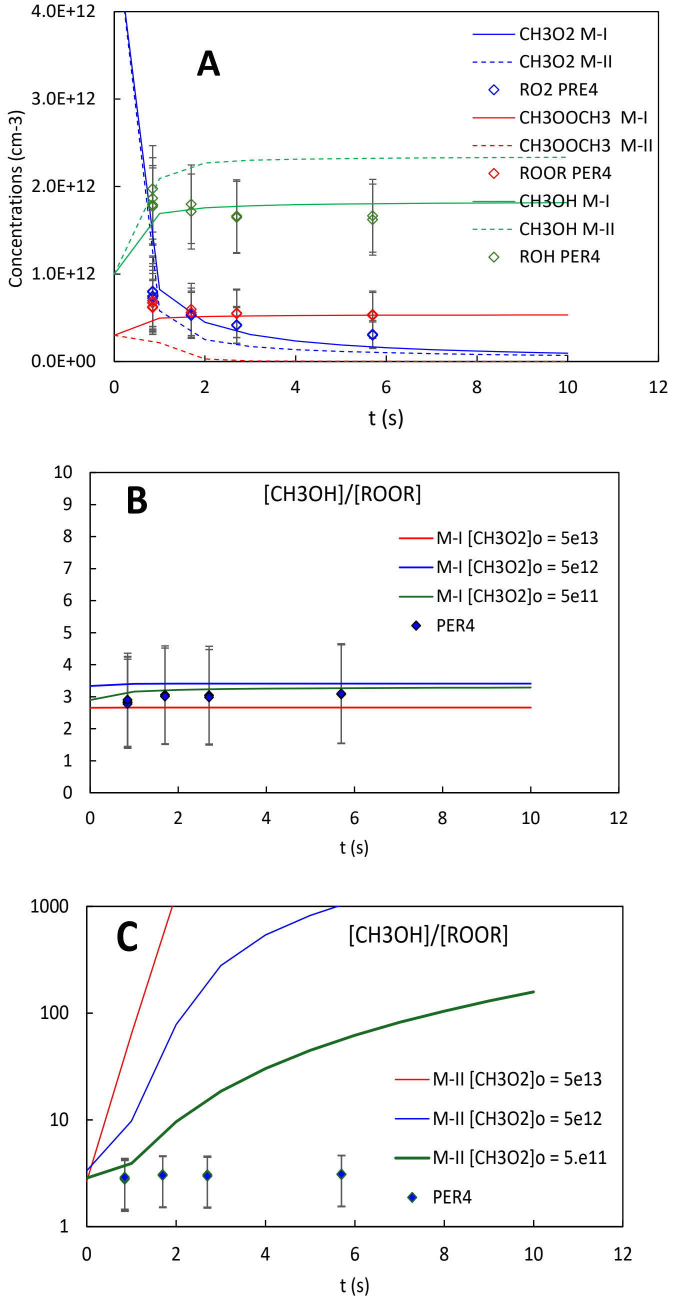

Figure S2: Comparison of mechanisms I and II for CH_3O_2. A) Time profiles simulated with $[CH_3O_2]o$ = 5e12 cm^{-3} and comparison with the experimental data of PER4 (open symbols); B) and C) evolution of the ratio R = $[CH_3OH]$ / $[H_3COOCH_3]$ in mechanism I and II for $[CH_3O_2]_o$ = 5e11 to 5e13 cm^{-3} and comparison with the ratio in experiment PER4 (blue symbols)

Table S4: Reactions and rate coefficients in Mechanisms I and II for t-$C_4H_9O_2$.

Mechanism I	Mechanism II
$RO_2 + RO_2 \rightarrow 2\ RO + O_2$ (1b$_1$)	$RO_2 + RO_2 \rightarrow ROOR + O_2$ (16)
$RO_2 + RO_2 \rightarrow ROOR + O_2$ (1c1)	$ROOR \rightarrow 2\ RO + O_2$ (17)
$RO \rightarrow CH_3COCH_3$ (13)	$RO \rightarrow CH_3COCH_3$ (13)
$CH_3 + O_2 \rightarrow CH_3O_2$ (14)	$CH_3 + O_2 \rightarrow CH_3O_2$ (14)
$RO_2 + CH_3O_2 \rightarrow RO + HCHO + O_2$ (1b2)	$RO_2 + CH_3O_2 \rightarrow RO + HCHO + O_2$ (1b2)
$CH_3O_2 + CH_3O_2 \rightarrow 2\ CH_3O + O_2$ (1b3)	$CH_3O_2 + CH_3O_2 \rightarrow 2\ CH_3O + O_2$ (1b3)
$CH_3O + O_2 \rightarrow HCHO + HO_2$ (10)	$CH_3O + O_2 \rightarrow HCHO + HO_2$ (10)
$CH_3O_2 + HO_2 \rightarrow CH_3OOH + O_2$ (11)	$CH_3O_2 + HO_2 \rightarrow CH_3OOH + O_2$ (11)
$RO_2 + HO_2 \rightarrow ROOH + O_2$ (11)	$RO_2 + HO_2 \rightarrow ROOH + O_2$ (11)
$HO_2 + HO_2 \rightarrow H_2O_2 + O_2$ (12)	$HO_2 + HO_2 \rightarrow H_2O_2 + O_2$ (12)
$RO_2 + I \rightarrow RO_2I$ (13)	$RO_2 + I \rightarrow RO_2I$ (13)
$RO_2I + I \rightarrow RO_2 + I_2$ (14)	$RO_2I + I \rightarrow RO_2 + I_2$ (14)
$CH_3O_2 + I \rightarrow CH_3O_2I$ (13)	$CH_3O_2 + I \rightarrow CH_3O_2I$ (13)
$CH_3O_2I + I \rightarrow CH_3O_2 + I_2$ (14)	$CH_3O_2I + I \rightarrow CH_3O_2 + I_2$ (14)
$HO_2 + I \rightarrow HI + O_2$ (15)	$HO_2 + I \rightarrow HI + O_2$ (15)
$k_{1b1} = 1.05 \times 10^{-17}$ s^{-1} cm^{-3} [a]	$k_{16} = 2.1 \times 10^{-17}$ s^{-1} cm^{-3} [1]
$k_{1c1} = 1.05 \times 10^{-17}$ s^{-1} cm^{-3} [a]	$k_{17} = 1$ s^{-1}*
$k_{13} = 1 \times 10^{5}$ s^{-1} [1]	
$k_{14} = 8 \times 10^{6}$ s^{-1} [1]	
$k_{1b2} = 3 \times 10^{-15} \times 0.7 – 1$ s^{-1} cm^{-3} [b]	
$k_{1b3} = 1.3 \times 10^{-15}$ s^{-1} cm^{-3} [c]	
$k_{10} = 2 \times 10^{-15}$ s^{-1} cm^{-3} [1]	
$k_{11} = 5.2 \times 10^{-12}$ s^{-1} cm^{-3} [1]	
$k_{11} = 5.2 \times 10^{-12}$ s^{-1} cm^{-3} [1]	
$k_{12} = 1.6 \times 10^{-12}$ s^{-1} cm^{-3} [1]	
$k_{13} = 2 \times 10^{-11}$ s^{-1} cm^{-3} [2]	
$k_{14} = 1.5 \times 10^{-10}$ s^{-1} cm^{-3} [2]	
$k_{13} = 2 \times 10^{-11}$ s^{-1} cm^{-3} [2]	
$k_{14} = 1.5 \times 10^{-10}$ s^{-1} cm^{-3} [2]	
$k_{15} = 4 \times 10^{-14}$ s^{-1} cm^{-3} [1]	

[a] k_{1b1} and k_{1c1} are $k = 2.1 \times 10^{-17}$ s^{-1} cm^{-3} from Ref[1] multiplied by a branching ratio 0.5 for both channels, consistent with the results obtained in this work; [b] rate coefficient from Ref.[3] multiplied by a ratio between 0.7 and 1 (see text); [c] rate coefficient from Ref.[3] multiplied by an assumed ratio of 0.5; *estimated in this work.

Figure S3: Comparison of Mechanisms I and II for tert-$C_4H_9O_2$

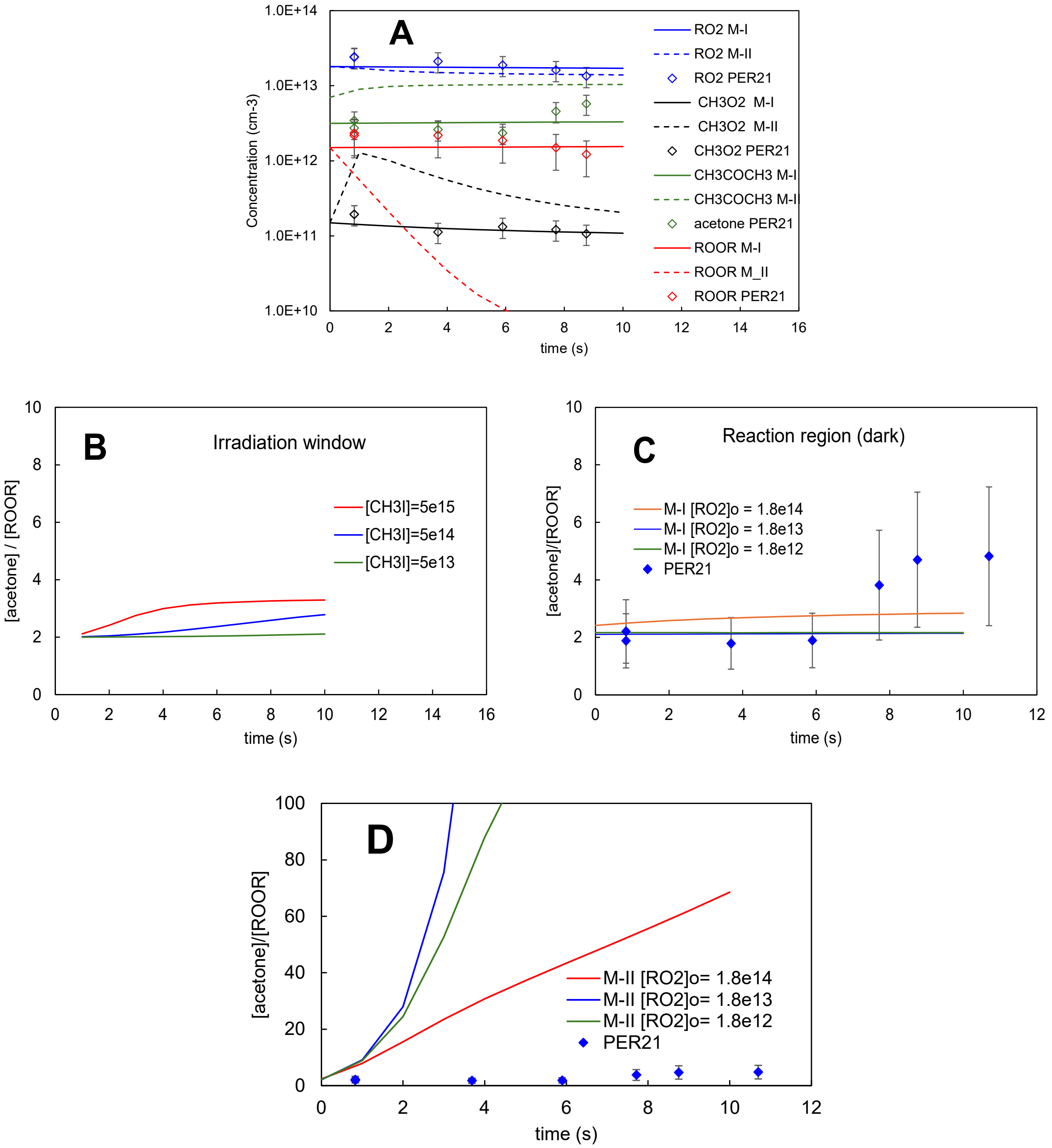

Figure S3: Comparison of Mechanisms I and II for tert-$C_4H_9O_2$. A) Time profiles simulated with $[RO_2]_o$ = 1.8e13 cm^{-3} and comparison with the experimental data of PER21 (open symbols); B) evolution of [acetone] / [ROOR] in the irradiation window in M-I (in PER21 irradiation time was 2.5 s); C) evolution of [acetone] / [ROOR] in the reaction region in M-I; D) evolution of [acetone] / [ROOR] in the reaction region in M-II.

Figure S4: Effects of side-reactions on the time profiles and product ratios.

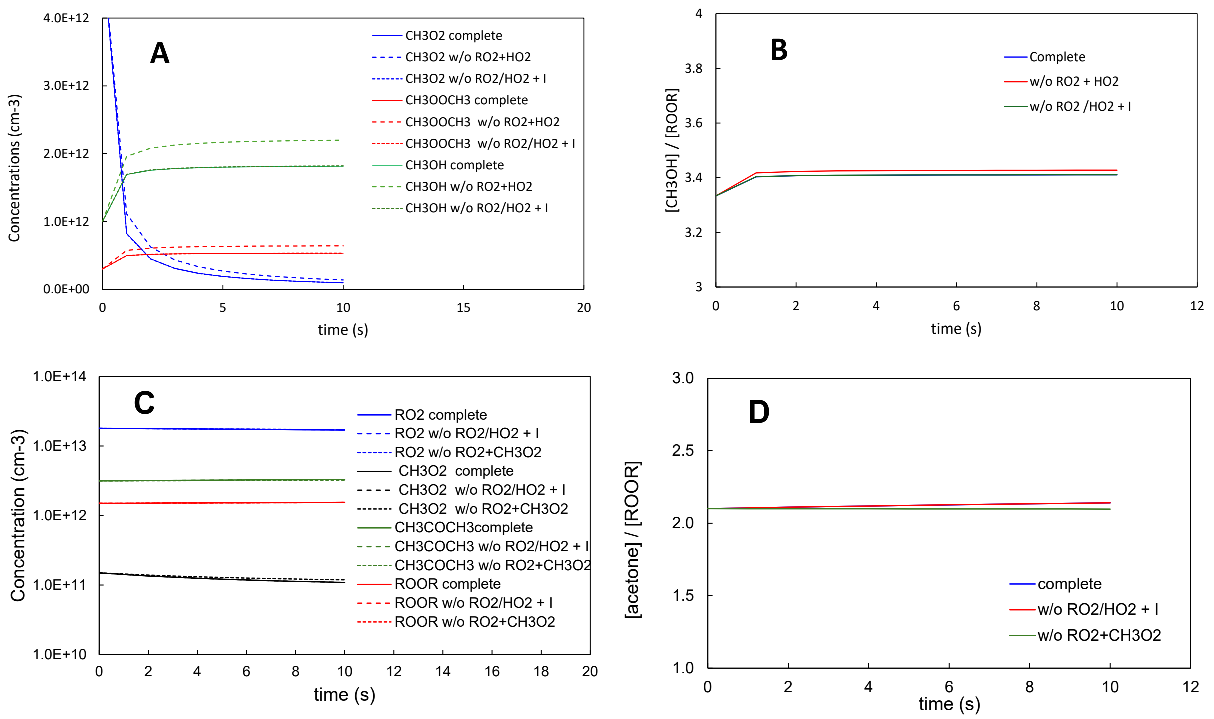

Figure S4: Effects of side-reactions on the time profiles and product ratios. A) and B) for CH_3O_2; C) and D) for t-$C_4H_9O_2$. The continuous lines are for the complete models (labelled "complete"). In A) and B) the dashed lines are where CH_3O_2 + HO_2 is suppressed and the dotted lines where CH_3O_2 + I and HO_2 + I are suppressed; In C) and D) the dashed lines are where RO_2 + I and CH_3O_2 + I are suppressed and the dotted lines are where RO_2 + CH_3O_2 is suppressed.

Figure S5: Evolution of the product ratios for different RO_2 in the experiments.

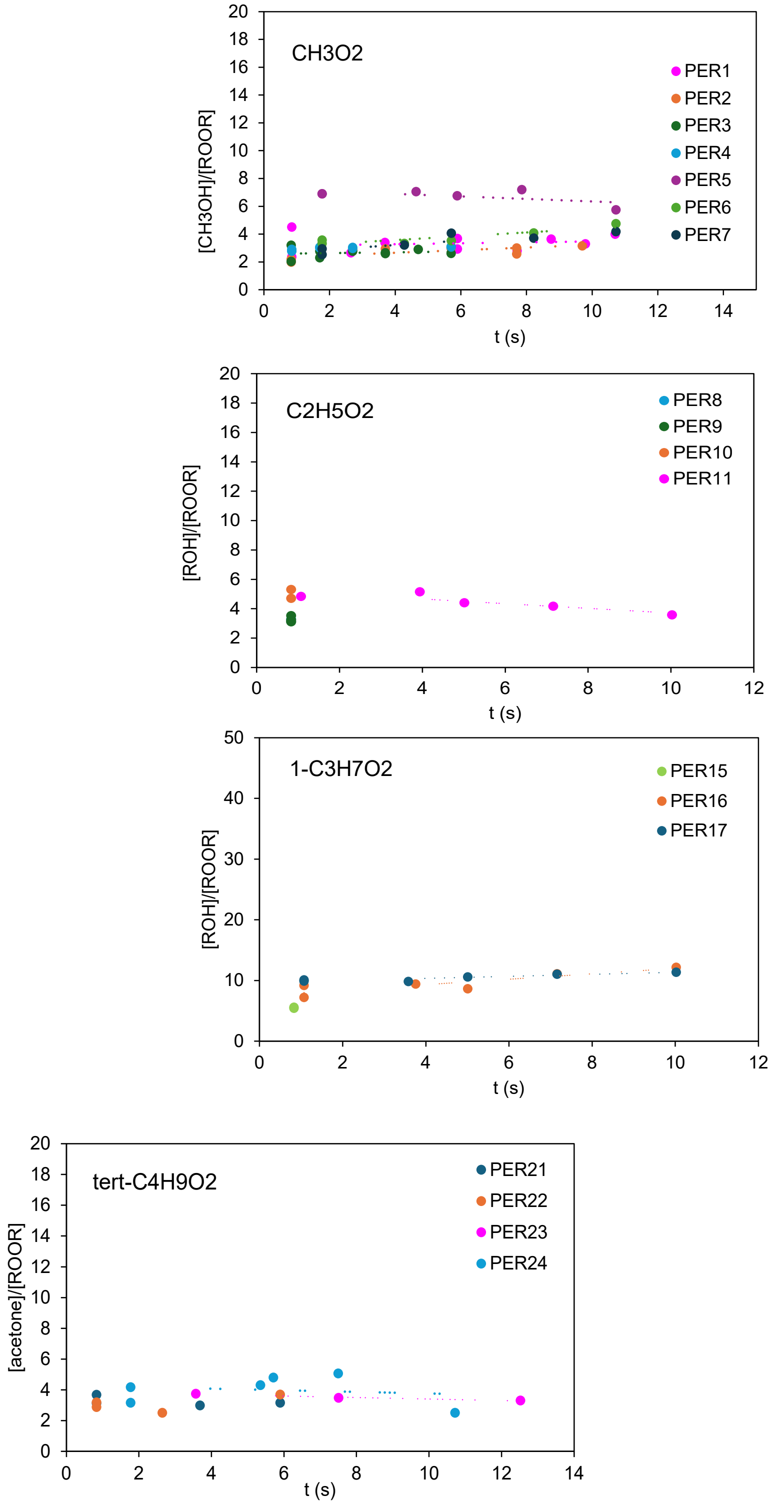

Section S2: Determination of γ from the observed product ratios and literature data for the other channels

For all RO_2 except t-$C_4H_9O_2$

For all the RO_2 studied in this work except t-$C_4H_9O_2$ the self-reaction proceeds through reaction (1) presented in the main text:

$$RO_2 + RO_2 \rightarrow ROH + R_{-H}{=}O + O_2 \qquad \alpha \text{ (1a)}$$
$$\rightarrow 2\,RO + O_2 \qquad \beta \text{ (1b)},$$
$$RO_2 + RO_2 \rightarrow ROOR + O_2 \qquad \gamma \text{ (1c)}.$$

The other reactions taking place in the system are

$$RO + O_2 \rightarrow R_{-H}{=}O + HO_2 \qquad (10)$$
$$RO_2 + HO_2 \rightarrow ROOH + O_2 \qquad (11).$$

Thus, no other reaction than channel (1a) produces the alcohol ROH and no other reaction than channel (1c) produces ROOR, so that

$$[ROH] / [ROOR] = R = \alpha/\gamma \quad \text{(Eq. 1)}$$

In each experiment, γ was thus determined from the measured ratio [ROH]/[ROOR] and the value for α from ref[1] or derived from the recommended value of β. It is important to note that Ref[1] recommends either k_1/k_{tot} for channel (1a) or k_2/k_{tot} for channel (1b), and that these ratios do not involve the assumption $k_1 + k_2 = k_{tot}$. In the present analysis, we took $\alpha = k_1/k_{tot}$ and $\beta = k_2/k_{tot}$. In Ref[1] $\alpha = k_1/k_{tot}$ is recommended only for $C_2H_5O_2$ (α = 0.3) and i-$C_3H_7O_2$ (α = 0.44), which were used directly to determine γ (Table 1 in Main text). For CH_3O_2, only $\beta = k_2/k_{tot}$ was recommended (β = 0.37). α was then derived from β and the observed product ratio R by

$$\alpha + \beta + \gamma = 1 \quad \text{(Eq. 2)}$$

replacing $\gamma = \alpha$ /R from Eq.(1)

$$\alpha\,(1 + 1/R) = 1 - \beta \quad \text{(Eq. 3)}$$

Thus $$\alpha = (1 - \beta) / (1+1/R) \quad \text{(Eq. 3)}.$$

The values of α thus obtained for CH_3O_2 with β = 0.37 and the observed ratios R were between 0.47 and 0.55 (Table 1 in main text).

For 1-$C_3H_7O_2$ and 1-$C_4H_9O_2$, for which no branching ratios are recommended, the same value of α as for $C_2H_5O_2$ was assumed (α = 0.3) in the analysis.

For t-$C_4H_9O_2$

For t-$C_4H_9O_2$, the self-reaction proceeds by two channels, (1b) and (1c), channel (1a) being unavailable because of the lack of H-atom on the C-atom carrying the radical group:

$$\text{t-}C_4H_9O_2 + \text{t-}C_4H_9O_2 \rightarrow \text{t-}C_4H_9O + \text{t-}C_4H_9O + O_2 \qquad \beta_1 \text{ (1b)}$$
$$\rightarrow \text{t-}C_4H_9O\text{ot-}C_4H_9O_2 + O_2 \quad \gamma_1 \text{ (1c)}$$

Unlike the alkoxy radicals for the other RO_2 discussed above, t-C_4H_9O decomposes into

$$\text{t-}C_4H_9O \rightarrow H_3CC(O)CH_3 + CH_3 \quad (13).$$

The branching ratio γ_1 for the channel producing the peroxide can thus be obtained from

$$[\text{acetone}]_1 / [ROOR] = 2\,\beta_1/\gamma_1 \qquad \text{(Eq. 4)}$$

Thus

$$\gamma_1 = 2\ \beta_1 \times [ROOR] / [acetone]_1 \quad \text{(Eq. 4)},$$

Where $[acetone]_1$ is the concentration of acetone resulting solely from reaction (1). However, the CH_3 radical produced in reaction (13) becomes CH_3O_2, which reacts rapidly with t-$C_4H_9O_2$, and faster than the self-reaction of t-$C_4H_9O_2$. This cross-reaction involves three channels:

$$\text{t-}C_4H_9O_2 + CH_3O_2 \rightarrow \text{t-}C_4H_9OH + HCHO + O_2 \quad \alpha_2\ (1a)$$
$$\rightarrow \text{t-}C_4H_9O + CH_3O + O_2 \quad \beta_2\ (1b)$$
$$\rightarrow \text{t-}C_4H_9OOCH_3O + O_2 \quad \gamma_2\ (1c),$$

And the radical t-C_4H_9O produced in channel (1b) produces an additional acetone molecule by reaction (13).

Therefore, to determine γ_1, it is necessary to evaluate $[acetone]_1$ compared to the total acetone concentration produced in the system and observed experimentally. This was made numerically, using the kinetic model in Table S4.3 (Mechanism I) and typical concentrations for the experiments. Since, to our knowledge, no value for the branching ratio β_2 in the cross- reaction has been reported, the latter was varied between 0.7 and 1 in the simulations, which did not make much difference in the results (see Fig. S6.1).

The results showed that, typically $[acetone]_1$ represented ~ 2/3 the total acetone concentration, $[acetone]_{tot}$. Thus, γ_1 was determined from the experiments by

$$\gamma_1 \sim 2\ \beta_1 \times 3/2 \times [ROOR] / [acetone]_{tot} =$$
$$= 3\ \beta_1 \times [ROOR] / [acetone]_{tot} = 3\ \beta_1 / R \quad \text{(Eq. 5)}.$$

Then, the condition $\beta_1 + \gamma_1 = 1$ gave an expression to determine γ_1 independently of β_1:

$$\gamma_1 = (3/R)/(1+3/R) \quad \text{(Eq. 6)}.$$

The values obtained in the experiments are presented in Table 1 in the main text. The average value of R ~ 3.45 measured in the experiments thus allowed to estimate β_1 ~ 0.534, which is very different from the value of 1 assumed in the literature.

Figure S6: Results of the kinetic simulations.

Top: time profile for the main compounds simulated with Mechanism I and β_2 = 1 in the cross-reaction between t-$C_4H_9O_2$ and CH_3O_2 and comparison with the experimental data in PER24 (symbols); Bottom: Simulation of the ratio $[\text{acetone}]_{\text{total}}/[\text{acetone}]_1$ for β_2 = 0.7 and 1.

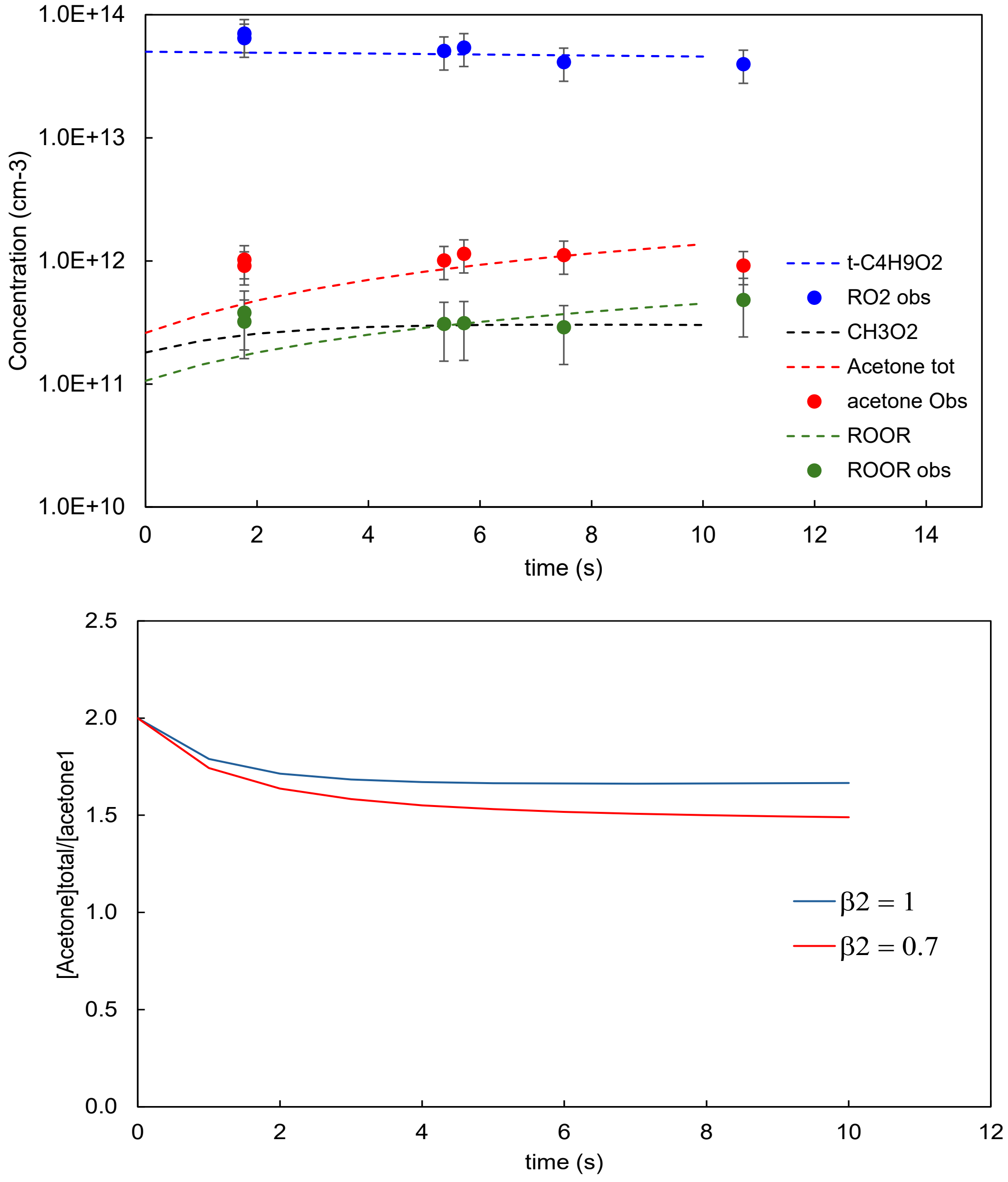

References

1. R. Atkinson, D. L. Baulch, R. A. Cox, J. N. Crowley, R. F. Hampson, R. G. Hynes, M. E. Jenkin, M. J. Rossi and J. Troe, *Atmos. Chem. Phys.*, 2006, **6**, 3625-4055.
2. T. J. Dillon, M. E. Tucceri and J. N. Crowley, *Phys. Chem. Chem. Phys.*, 2006, **8**, 5185-5198.
3. B. Nozière and D. R. Hanson, *J. Phys. Chem. A*, 2017, **121**, 8453-8464.
